# William Stokes: His Life and Work (1804—1878)

**Published:** 1900-03

**Authors:** 


**William Stokes: his Life and Work (1804—1878).** By his Son, WILLIAM STOKES. Pp. 256. London: T. Fisher Unwin. 1898.

The fourth volume of the "Masters of Medicine" series, written by Sir William Stokes, Surgeon-in-Ordinary to the Queen in Ireland, has as its object "to convey the impression of the wide sympathies, many-sided nature, and greatness of the character portrayed, not so much by description, as by a simple record of the scientific work achieved by him, along with what may be gathered from his addresses and occasional letters, of his exalted standard of professional honour, and his love and appreciation of the beautiful in Nature and Art." Five pages of bibliography conclude the volume. The work for which Stokes will be long remembered was *Diseases of the Heart and Aorta*, published in 1854, in which he insisted on the utility of a system of graduated muscular exercises in the treatment of debility of the heart.

But Stokes was more than an accomplished physician. He was a man of much general culture, and in almost any branch of letters or science to which he might have devoted his life-work he would have been distinguished. A very striking and beautiful portrait, after Sir Frederick Burton, adorns the volume.

